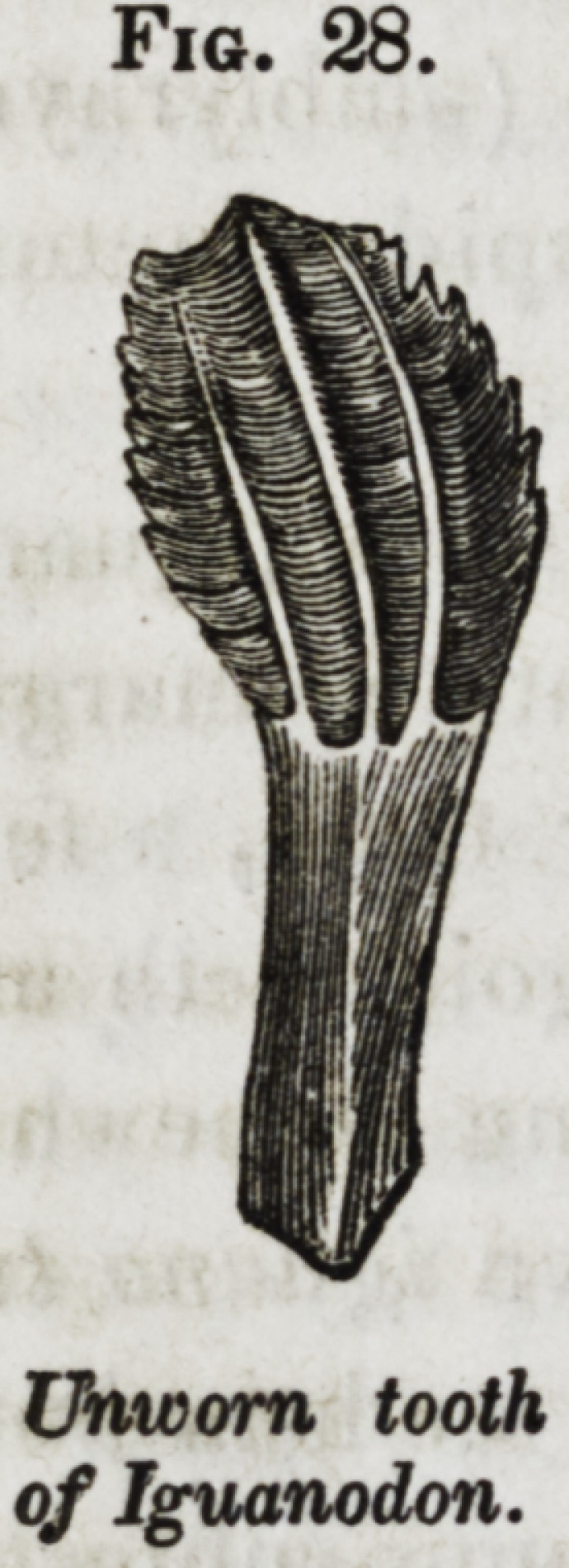# Teeth—Comparative Anatomy

**Published:** 1852-04

**Authors:** 


### SELECTED ARTICLES.

### ARTICLE VII.

### *Teeth—Comparative Anatomy.*

(Continued from page 305.)

I PROCEED now to briefly point out the leading characteristics of the teeth in the different classes of the vertebrate animals.

### *Dental System of Fishes.*

The teeth of fishes, whether we study them in regard to their number, form, substance, structure, situation, or mode of attachment, offer a greater and more striking series of varieties than do those of any other class of animals.

As to *number*, they range from zero to countless quantities.

The lancelet, the ammocete, the sturgeon, the paddle-fish, and the whole order of *lophobranchii*, are edentulous. The myxinoids have a single pointed tooth on the roof of the mouth, (*fig.* 16, *a*,) and two serrated dental plates (*b*) on the tongue. The tench (vol. iii. p. 979, article Pisces) has a single grinding tooth on the occiput (*c*,) opposed to two dentigerous pharyngeal jaws below (*dd.*) In the lepidosiren a single maxillary dental plate (*fig.* 17, *a*,) is opposed to a single man-

Fig. 16.

*Myxine.* (*Muller.*)

Fig. 17.

*Lepidosiren.*

dibular bone (*b*,) and there are two small denticles on the nasal bone (*c.*) In the extinct sharks with crushing teeth, called *ceratodus* and *ctenodus*, the jaws were armed with four teeth, two above and two below. In the *chimæræ*, two mandibular teeth are opposed to four maxillary teeth. From this low point the number in different fishes is progressively multiplied, until, in the pike, the siluroids (*fig.* 18,) and many other fishes, the mouth becomes crowded with countless teeth.

With respect to *form*, I may first observe, that as organised beings withdraw themselves more and more, in their ascent in the scale of life, from the influence of the general polarising forces, so their parts progressively deviate from geometrical figures: it is only, therefore, in the lowest vertebrated class, that we find teeth in the form of perfect cubes, and of prisms or plates with three sides (*myletes*,) four sides (*scarus*,) five or six sides, *myliobates* (*fig.* 19.) The cone is the most common form in fishes: such teeth may be slender, sharp-pointed, and so minute, numerous, and closely aggregated, as to resemble the plush or pile of velvet; these are called "villiform teeth"

(*dentes villiformes, dents en velours ;**) all the teeth of the perch are of this kind; when the teeth are equally fine and numerous,

FIG. 18.

*Palatine bone and teeth* (*Silurus.*)

FIG. 19.

*Jaws and teeth* (*Myliobates.*)

but longer, they are called "ciliiform" (*dentes ciliiformes:*) when the teeth are similar to but rather stronger than these, they are called "setiform" (dentes setiformes, dentes en brosse) conical teeth, as close set and sharp pointed as the villiform teeth, but of larger size, and called "rasp teeth" (dentes raduliformes, dens en rape or en cardes, fig. 18;) the pike presents such teeth on the back part of the vomer; the teeth of the sheat-fish (silurus glanis) present all the gradations between the villiform and raduliform types. Setiform teeth are common in the fishes thence called chætodonts;† in the genus *citharina* they bifurcate at their free extremities; in the genus *platax* they end there in three diverging points (fig. 20,) and the cone here merge into the long and slender cylinder. Sometimes the

* The French terms are those used by Cuvier and Valenciennes in their great "Historie des Poissons," 4to.

cone is compressed into a slender trenchant blade: and this may be pointed and recurved, as in the *muræna*; or barbed, as in *trichiurus*, and some other scomberoids; or it may be bent upon itself, like a tenterhook, as in the fishes thence called goniodonts.* In the bonito may be perceived a progressive thickening of the base of the conical teeth: and this being combined in other predatory fishes with increased size and recurved direction, they then resemble the laniary or canine teeth of carniverous quadrupeds, as we see in the large teeth of the pike, in the lophius, and in certain sharks.

The anterior diverging grappling teeth of the wolf-fish, form stronger cones; and by progressive blunting, flattening, and expansion of the apex, observable in different fishes, the cone gradually changes to the thick and short cylinder, such as is seen in the back teeth of the wolf-fish, and in similar grinding and crushing teeth in other genera, whether feeders on sea-weeds, or crustaceous and testaceous animals. The grinding surface of these short cylindrical teeth, may be convex, as in the sheep's-head fish (sargus;) or flattened, as in the pharyngeal teeth of the wrasse (labrus.) Sometimes the hemispheric teeth are so numerous, and spread over so broad a surface, as to resemble a pavement, as in the pharyngeal bones of the wrasse or rockfish (labrus, fig. 21;) or they may be so small,

FIG. 20.

*Mandibular teeth, magnified (Platax.)*

FIG. 21.

*Inferior pharyngeal bone and teeth (Labrus.)*

*Γωνία, an angle; οδους, a tooth.

as well as numerous (dentes graniformes,) as to give a granulated surface to the part of the mouth to which they are attached (premaxillaries) of cossyphus. A progressive increase of the transverse over the vertical diameter, may be traced in the molar teeth of different fishes, and sometimes in those of the same individual, as in labrus, (fig. 21,) until the cylindrical form is exchanged for that of the depressed plate. Such dental plates (dentes lamelliformes) may be found, not only circular, but elliptical, oval, semilunar, sigmoid, oblong, or even square, hexagonal, pentagonal, or triangular; and the grinding surface may present various and beautiful kinds of sculpturing. The broadest and thinnest lamelliform teeth are those that form the complex grinding tubercle of the diodon. The front teeth of the flounder and sargus, present the form of compressed plates, at least in the crown, and are true *dentes incisivi.* Numerous wedge-shaped dental plates (dentes cuneati) are set vertically in the upper pharyngeal bones of the parrot-fish (scarus, fig. 22.) A thin lamella, slightly curved like a finger-nail, is the singular form of a tooth in an extinct genus of fishes, which I have thence called *petalodus.*

Fig. 22.

*Superior pharyngeal bones and teeth* (*Scarus.*)

Sometimes the incisive form of tooth is notched in the middle of the cutting edge, as in sargus unimaculatus. Sometimes the edge of the crown is trilobate (aplodactylus, fig. 23.) Sometimes it is made quinquelobate by a double notch on each side of the large middle lobe (boops.) In the formidable sea-pike (sphyræna barracuda) the crown of each tooth, large and small, is produced into a compressed and sharp point, and resembles a lancet. Sometimes the edges of such lancet-shaped

Fig. 23.

*Front teeth of Aplodactylus.*

teeth are finely serrated, as in *priodon*, and the great sharks of the genus *carcharias*, the fossil teeth of which indicate a species (carch. megalodon) sixty or seventy feet in length.

The lanceted form is exchanged for the stronger spear-shaped tooth in the sharks of the genus *lamna;* and in the allied great extinct *otodus*, as in the small porbeagle, similarly shaped, but stronger, piercing and cutting teeth were complicated by one or more accessory compressed cusps on each side their base, like the Malay crease.

With respect to situation, the teeth in sharks and rays, are limited to the bones (maxillary and mandibular,) which form the anterior aperture of the mouth : in the carp and other cyprinoids, the teeth are confined to the bones (pharyngeal and basi-occipital) which circumscribe the posterior aperture of the mouth. The wrasses (labrus) and the parrot-fishes (scarus) have teeth on the pre-maxillary and pre-mandibular, as well as on the upper and lower pharyngeals; both the anterior and posterior apertures of the mouth being thus provided with instruments for seizing, dividing, or comminuting the food, the grinders being situated at the pharynx. In most fishes teeth are developed also in the intermediate parts of the oral cavity, as on the palatines, the vomer, the hyoid bones, the branchial arches; and, though less commonly, on the pterygoids, the entopterygoids, the sphenoids, and even on the nasal bone, (fig. 17, *c*.) It is very rare to find teeth developed on the true superior maxillary bones; but the herring and salmon tribes, some of the ganoid fishes, and the great sudis, are examples of this approach to the higher vertebrata. Among the anomalous positions of teeth may be cited, besides the occipital alveolus of the carp, the marginal alveoli of the prolonged, depressed, well ossified rostrum of the saw-fish, *pristis*. In the lampreys and in helostomus (an osseous fish,) most of the teeth are attached to the lips. Lastly, it is peculiar to the class *pisces*, amongst vertebrata, to offer examples of teeth developed in the median line of the mouth, as in the palate of the myxines (fig. 16, *a*,) or crossing the symphysis of the jaw, as in *notidanus*, *sycmnus*, and *myliobates*.

Nor is the mode less varied than the place of attachment. The teeth of *lophius*, *pæcilia*, *anableps*, are always movable. In most fishes they are anchylosed to the jaws by continuous ossification from the base of the dental pulp ; the histological transition being more or less gradual from the structure of the tooth to that of the bone. Sometimes we find, not the base, but one side of the tooth anchylosed to the alveolar border of the jaw ; and the teeth oppose each other by their sides instead of their summits (scarus ;) in *pimelodus*, however, where the teeth are thus attached, the crown is bent down in the upper teeth, and bent up in the lower ones, at right angles to the fang, so that they oppose each other by the normal surfaces. Certain teeth of recent and fossil cartilaginous fishes have their base divided into processes like fangs, but these serve for the attachment of ligaments, and are not set in bony sockets like the true fangs or roots of the teeth of the mammalia. Some sharks have two divaricating fangs : some fossil teeth referred to my genus *petalodus* by Agassiz, with the specific name "*radicans*," have the base divided into several fangs or processes, indicating a generic distinction. The base of anchylosed teeth is, at first, attached to the jaw-bone, by ligament ; and in the cod-fish, wolf-fish, and some other species, as calcification of the tooth progresses towards its base, the subjacent portion of the jaw-bone receives a stimulus, and develops a process corresponding in size and form with the base of the tooth : for some time a thin layer of ligamentous substance intervenes, but anchylosis usually takes place to a greater or less extent before the tooth is shed. Most of the teeth of the lophius retain the primitive ligamentous connection ; the ligaments of the large internal or posterior teeth of the upper and lower jaws, radiate on the corresponding sides of the bone, the base of the tooth resting on a conformable alveolar process. The ligaments do not permit the tooth to be bent outwards beyond the vertical position, but yield to pressure in the contrary direction, by which the point of the tooth may be directed towards the back of the mouth ; the instant, however, that the pressure is remitted, the tooth returns through the elasticity of the bent ligaments, as by the action of a spring, into its usual erect positions (vol. iii, p. 978, article Pisces;) the deglutition of the prey of this voracious fish is thus facilitated, and its escape prevented. The broad and generally bifurcate bony base of the teeth of sharks is attached by ligament to the semiossified crust of the cartilaginous jaws; but they have no power of erecting or depressing the teeth at will. The small and closely crowded teeth of rays, are also connected by ligaments to the subjacent maxillary and mandibular membranes. The broad tesselated teeth of the *mylobates*, have their attached surface longitudinally grooved to afford them better hold-fast, and the sides of the contiguous teeth are articulated together by serrated or finely undulating sutures, a structure unique in dental organization. The teeth of the *sphyræna* are examples of the ordinary implantation in sockets, with the addition of a slight anchylosis of the base of the fully-formed tooth with the alveolar parietes; and the compressed rostral teeth of the saw-fish are deeply implanted in sockets. In the latter, the hind margin of their base is grooved, and a correspoding ridge from the back part of the socket fits into the groove, and gives additional fixation to the tooth. Some implanted teeth in the present class, have their hollow base further supported, like the claws of the feline tribe, upon a bony process arising from the base of the socket; the incisors of the balistes, *e. g.* afford an example of this double or reciprocal gomphosis. In fact, the whole of this part of the organization of fishes is replete with beautiful instances of design, and instructive illustrations of animal mechanics. The vertical section of a pharyngeal jaw and teeth of the wrasse (labrus) would afford the architect a model of a dome of unusual strength, and so supported as to relieve from pressure, the floor of a vaulted chamber beneath. The base of the dome-shaped tooth is slightly contracted, and is implanted in a shallow circular cavity; the rounded margin of which is adapted to a circular groove in the contracted part of the base; the margin of the tooth which immediately transmits the pressure of the bone, is strengthened by an inwardly projecting convex ridge.

The masonry of this inner buttress, and of the dome itself, is composed of hollow columns, every one of which is placed so as best to resist or transmit in the due direction the external pressure. The floor of the alveolus is thus relieved from the office of sustaining the tooth: it forms, in fact, the roof of a lower vault, in which the germ of a successional tooth is in course of development; had the crushing tooth in use, rested, as in the wolf-fish, by the whole of its base upon the alveolus, the supporting plate, gradually undermined by the growth of the new tooth, must have given way, and been forced upon the subjacent delicate and highly vascular and sensitive matrix of the half-formed tooth. But the superincumbent pressure is exclusively sustained by the border of the alveolus, whence it is transferred to the walls dividing the vaulted cavities containing the germs of the new teeth; the roofs of these cavities yield to the absorbent process consequent on the growth of the new teeth without materially weakening the attachment of the old teeth, and without the new teeth being subjected to any pressure, until their growth is sufficiently advanced to enable them to bear it with safety; by this time the sustaining borders of the old alveolus are undermined, and the old worn-down tooth is shed.

The singular and powerfully developed dental system of the wolf-fish (*anarrhichas lupus*) has been a subject of interest to many anatomists. The general character and physiological relations of the teeth in this species had not escaped the attention of Hunter. In his paper on the gillaroo trout, read before the Royal Society in 1774, he observes that "the teeth of fishes which subsist chiefly on animal matter must vary according as their food may be common salt fish, or shell-fish." "Such fish as live on the first kind have, like the carnivorous quadrupeds and birds, no apparatus for mastication, their teeth being intended merely for catching the food and fitting it to be swallowed. But the shells of the second kind of food render some degree of masticatory power necessary to fit it for its passage either into the stomach or through the intestines: and accordingly we find in certain fish a structure suited to the purpose.

Thus the mouth of the wolf-fish is almost paved with teeth, by means of which it can break shells to pieces, and fit them for the œsophagus of the fish, and so effectually disengage the food from them, that though it lives upon such hard food, the stomach does not differ from that of other fish."

But in order to secure the capture of the shell-fish, the teeth of the wolf-fish are not all crushers; some present the laniary type, with the apices more or less recurved and blunted by use, and consist of strong cones spread abroad, like grappling hooks, at the anterior part of the mouth.

The premaxillary teeth are all conical, and arranged in two rows; there are two, three, or four in the exterior row, at the mesial half of the bone, which are the largest; and from six to eight smaller teeth are irregularly arranged behind. There are three large, strong, diverging laniaries at the anterior end of each premandibular bone, and immediately behind these an irregular number of shorter and smaller conical teeth, which gradually exchange this form for that of large obtuse tubercles; these extend backwards, in a double alternate series, along a great part of the alveolar border of the bone, and terminated by two or three smaller teeth in a single row, the last of which again presents the conical form. Each palatine bone supports a double row of teeth, the outer ones being conical and straight, and from four to six in number, the inner ones, two, three or four in number, and tuberculate. I have seen a specimen where the inner row was wanting on one side. The lower surface of the vomer is covered by a double irregularly alternate series of the same kind of large tuberculate crushing teeth as those at the middle of the premandibular. All the teeth are anchylosed to more or less developed alveolar eminences, like the anterior teeth of the lophius. The periphery of the expanded circular base of the large anterior grappling teeth is divided into processes indicative of the original ligamentous fasciculi at the base of the pulp by the ossification of which their anchylosis is effected.

When such anchylosed teeth and the supporting bone are divided by a vertical section, as in fig. 2, pl. 66, of my "Odontography," there may be generally discerned a faint transverse line indicating the original separation between the tooth and the bone, and more clearly defining the dental from the osseous structure, than in the anchylosed teeth of other fishes. From the enormous development of the muscles of the jaws, and the strength of the shells of the whelks and other testacea which are cracked and crushed by the teeth, their fracture and displacement must obviously be no unfrequent occurrence; and most specimens of the jaws of the wolf-fish exhibit some of the teeth either separated at this line of imperfect anchylosis, or, more rarely, broken off above the base, or, still more rarely, detached by fracture of the supporting osseous aveolar process.

With regard to the substance of the teeth of fishes, the modifications of dentine, called vaso-dentine and osteo-dentine, predominate much more than in the higher vertebrata; and they thus more closely resemble the bones which support them. There is, however, great diversity in respect of substance. The teeth of most of the chætodonts are flexible, elastic, and composed of a yellowish subtransparent albuminous tissue; such, likewise, are the labial teeth of the helostome, the premaxillary and mandibular teeth of the goniodonts, and of that percoid genus thence called trichodon. In the cyclostomes, the teeth consist of a denser albuminous substance. The upper pharyngeal molar of the carp consists of a peculiar brown and semi-transparent tissue, hardened by salts of lime and magnesia. The teeth of the flying-fish, (exocætus,) and sucking-fish, (remora,) consist of osteo-dentine. In many fishes, e. g. the acanthurus, sphyræna, and certain sharks (lamna,) a base, or body of osteo-dentine is coated by a layer of true dentine, but of unusual hardness, like enamel: in prionodon this hard tissue predominates. In the labrus the pharyngeal crushing teeth consist wholly of hard, or unvascular dentine. In most pycnodonts and cestracionts, and many other fishes, the body of the tooth consists of ordinary unvascular dentine, covered by a modification of that tissue which I have called "vitro-dentine" from its clear, polished, enamel-like character; but this is not enamel, nor the product of a distinct organ from the dentinal pulp; it differs from ordinary dentine in the greater proportion of the mineral particles, their more minute diffusion through the gelatinous basis, in the straighter course and more minute size of the calcigerous tribes; it results from the calcification of the external layer of the dentinal pulp, and is the first part of tooth which is formed. In sargus and balistes the body of the tooth consists of true dentine, and the crown is covered by a thick layer of a denser tissue, developed by a distinct organ, and differing from the "enamel" of higher animals only in the more complicated and organised mode of deposition of the earthy salts. The ossification of the capsule of the complex matrix of these teeth covers the enamel with a thin coating of "cement." In the pharyngeal teeth of the scarus a fourth substance is added by the ossification of the base of the pulp after its summit and periphery have been converted into hard dentine; and the teeth, thus composed of cement, enamel, dentine and osteo-dentine, are the most complex in regard to their substance that have yet been discovered in the animal kingdom.

The tubes which convey the capillary vessels through the substance of the osteo- and vaso-dentine of the teeth of the fishes* were early recognised, on account of their comparatively large size; as by André, e. g. in the teeth of acanthurus, and by Cuvier and Von Born in the teeth of the wolf-fish and other species. Leeuwenhoek had also detected the much finer tubes of the peripheral dentine of the teeth of the haddock. These "dentinal tubuli" are given off from the parietes of the vascular canals, and bend, divide, and subdivide rapidly in the hard basis-tissue of the interspaces of those canals in osteo-dentine; the dental tubuli alone are found in true dentine, and they have a straighter and more parallel course,

* The vaso-dentine of pristis and myliobates is like that of the teeth of the cape ant-eater (orycteropus:) the vaso-dentine of the psammodonts resembles that which forms the base of the tooth of the sloth and megatherium: the vaso-dentine of mammals differs from the osteo-dentine in the absence of the radiated "Purkinjian," cells.

usually at right angles to the outer surface of the dentine. Those conical teeth which, when fully formed, consist wholly, or in great part, of osteo-dentine or vaso-dentine, always first appear with an apex of hard or true dentine. In some fishes the simple central basal pulp-cavity of such teeth, instead of breaking up into irregular or parallel canals, sends out a series of vertical plates from its periphery, which, when calcified, give a fluted character to the base of the tooth, e. g. in lepidosteus oxyurus.* Sometimes such radiating vertical basal plates of dentine are wavy in their course, and send off narrow processes from their sides; and, as a thin layer of the outer capsule interdigitates with the outstanding plates of the dentinal pulp, and becomes co-calcified with them, a transverse section of such a tooth presents a series of interblended wavy, or labyrinthic tracks of thick dentine radiating from the center, and of thin cement converging to the center of the tooth.† An analogous but more complicated structure obtains when the radiating, wavy, vertical plates of dentine dichotomise, and give off from their sides, throughout their course, numerous branch plates and processes, which are traversed by medullary sinuses and canals with their peripheral terminations dilated, and becoming the centers of lobes or columns of hard dentine. The transverse section of such teeth gives the appearance of branches of a tree, with leaf-stalks and leaves, radiating from the central pulp-cavity to the circumference of the tooth; and

* Wyman, American Journal of Natural Sciences, Oct. 1843. Cuvier has given an accurate view of the plaited structure of the base of the wolf-fish's teeth in pl. 32, fig. 7, of his Leçons d'Anatomie Comparée, 1805; where it is described at the base of the osseous tubercle, which supports the true tooth.

† This remarkable structure attains its highest complication and forms the largest proportion of the tooth in the gigantic extinct labyrinthodont batrachia, and from which, therefore, I have taken the illustrations of that complex modification of dental structure. I had discovered in 1841, the more simple modification of this structure "at the base of the tooth in a few fishes" (Geol. Trans., 2d series, vol. vi., p. 507,) but had not then seen so complex an example in that class as Dr. Wyman and M. Agassiz (Recherches sur les Poissons Fossiles, "Sauroides" 1843) subsequently described and figured, in teeth of the genus lepidosteus.

I have called the fossil fish in which this structure was first detected, dendrodus.

Thus, with reference to the main and fundamental tissue of teeth; we find not fewer than six leading modifications in fishes; hard or true dentine, (sparoids, labroids, lophius, balistes, pycnodonts, prionodon, sphyræna, megalichthys, rhizodus, diodon, scarus;) osteodentine, (cestracion, acrodus, lepidosiren, ctenodus, hybodus, percoids, sciænoids, cottoids, gobioids, and many others;) vaso-dentine, (psammodus, chimæroids, pristis, myliobates;) plici-dentine, (lophius, holoptychius, lepidosteus oxyurus, at the base of the teeth;) labyrinthodentine, (lepidosteus, platyrhinus, bothriolepis;) and dendrodentine, (dendrodus;) besides the compound teeth of the scarus and diodon.

One structural modification may prevail in some teeth, another in other teeth of the same fish; and two or more modifications may be present in the same tooth, arising from changes in the process of calcification and a persistency of portions or processes of the primitive vascular pulp or matrix of the dentine.

The dense covering of the beak-like jaws of the parrot-fishes, (scari,) consists of a stratum of prismatic denticles, standing almost vertically to the external surface of the jaw-bone. An account of the structure and development of this peculiar armature of the jaws is abridged from my "Odontography" p. p. 112—116, in the article PISCES, vol. iii. p. 979. It is peculiarly adapted to the habits and exigencies of a tribe of fishes which browse upon the lithophytes that clothe, as with a richly tinted carpet, the bottom of the sea, just as the ruminant quadrupeds crop the herbage of the dry land.

The irritable bodies of the gelatinous polypes which constitute the food of these fishes retract, when touched, into their star-shaped stony shells, and the scari consequently require a dental apparatus strong enough to break off or scoop out these calcareous recesses. The jaws are, therefore, prominent, short, and stout, and the exposed portions of the premaxillaries and premandibulars are encased by complicated dental covering.

The polypes and their cells are reduced to a pulp by the action of the pharyngeal jaws and teeth, that close the posterior aperture of the mouth.

The superior dentigerous pharyngeals, *figs*. 15 and 22, present the form of an elongated, vertical, inequilateral, triangular plate; the upper and posterior margin is sharp and concave; the upper and anterior margin forms a thickened articular surface, convex from side to side, and playing in a corresponding groove or concavity upon the base of the skull; the inferior boundary of the triangle is the longest, and also the broadest; it is convex in the antero-posterior direction, and flat from side to side. It is on this surface, that the teeth are implanted, and in most species they form two rows; the outer one consisting of very small, the inner one of large dental plates, which are set nearly transversly across the lower surface of the pharyngeal bone, and are in close apposition, one behind the other: their internal angles are produced beyond the margin of the bone, and interlock with those of the adjoining bone when the pharyngeals are in their natural position; the smaller denticles of the outer row are set in the external interspaces of those of the inner row.

The single inferior pharyngeal bone consists principally of an oblong dentigerous plate, of the form represented in *fig*. 3, pl. 51, of my "Odontography;" its breadth somewhat exceeds that of the conjoined dentigerous surface of the pharyngeals above, and it is excavated to correspond with their convexity. This dentigerous plate is principally supported by a strong, slightly curved, transverse, osseous bar, the extremities of which expand into thick obtuse processes for the implantation of the triturating muscles. A longitudinal crest is continued downwards and forwards from the middle line of the inferior pharyngeal plate, anterior to the transverse bar, to which the protractor muscles are attached.

A longitudinal row of small oval teeth alternating with the large lamelliform teeth, like those of the superior pharyngeals, bounds the dentigerous plate on each side; the intermediate space is occupied exclusively by the larger lamelliform or wedge-shaped teeth, set vertically in the bone, and arranged transversely in alternate and pretty close set series.

The dental plates are developed in wide and deep cavities in the substance of the posterior part of the lower, and of the anterior part of the upper pharyngeal bones. Each denticle is developed in its proper capsule, which contains an enamel-forming pulp and a dentinal pulp, in close cohesion with each other, and with the thin external capsule. The teeth exhibit progressive stages of formation, as they approach the posterior part of the upper and the anterior part of the lower pharyngeal bones: as their formation advances to completion they become soldered together by ossification of their respective capsules into one compound tooth, which soon becomes anchylosed by ossification of the dentinal pulp to the pharyngeal bone itself.

The dentine of the pharyngeal teeth of the scarus consists of calcigerous tubes, and a clear intermediate substance. The calcigerous tubes average a diameter of $\frac{1}{20,000}$ of an inch, and are separated by interspaces equal to twice their own diameter. The course of these tubes is shown in *fig.* 15, *d*, in which they are exposed by a vertical section through the middle of two of the superior denticles. They all, on leaving the pulp cavity, form a curve with the convexity turned towards the base of the tooth, and then bend slightly in the opposite direction; the sigmoid curve being most marked in the calcigerous tubes at the base of the denticles, whilst those towards the apex become longer and straighter. Besides the primary curvatures exemplified in the figure, each calcigerous tube is minutely undulated; it dichotomises three or four times near its termination, sends off many fine lateral branches into the clear uniting substance, and finally terminates in a series of minute cells and inosculating loops at the line of junction with the enamel.

This substance (fig. 15, *e.)* is as thick as the dentine, and consists of a similar combination of minute tubes, and a clear connecting substance. The tubes may be described as commencing from the peripheral surface of the tooth to which they stand at right angles, and, having proceeded parallel to each other halfway towards the dentine, they then began to divide and subdivide, the branches crossing each other obliquely, and finally terminating in the cellular boundary between the enamel and dentine.

The teeth which present this complex structure are successively developed at one extremity of the bone, in proportion as they are worn away at the other; not, however, as Cuvier describes, from behind forwards, in both upper and lower pharyngeal bones, but in opposite directions in the opposite bones, the course of succession being from before backwards in the upper, and from behind forwards in the lower pharyngeal bones. In the progress of the attrition to which they are subjected, the thin coat of cement resulting from the ossification of the capsule is first removed from that apex of the tooth, then the enamel constituting the apex, next the dentine, and finally, the coarse central cellular bone, supporting the hollow wedge-shaped tooth; and thus is produced a triturating surface of four different substances of different degrees of density. The enamel, being the densest element, appears in the form of elliptical transverse ridges, inclosing the dentine and central bone; and external to the enamel is the cement which binds together the different denticles.

There is a close analogy between the dental mass of the scarus and the complicated grinders of the elephant, both in form, structure, and in the reproduction of the component denticles in horizontal succession. But in the fish, the complexity of the triturating surface is greater than in the mammal, since, from the mode in which the wedge-shaped denticles of the scarus are implanted upon, and anchylosed to, the processes of the supporting bone, this likewise enters into the formation of the masticatory surface when the tooth is worn down to a certain point.

The proof of the efficacy of the complex masticatory apparatus above described is afforded by the contents of the alimentary canal of the scari. Mr. Charles Darwin, the accomplished naturalist and geologist, who accompanied Captain Fitzroy, R. N., in the circumnavigatory voyage of the "Beagle,"

dissected several parrot-fishes soon after they were caught, and found the intestines laden with nearly pure chalk, such being the nature of their excrements; whence he ranks these fishes among the geological agents to which is assigned the office of converting the skeletons of the lithophytes into chalk.

*Development.*—As might have been anticipated from the discovery of the varied and predominating vascular organization in the teeth of fishes, and the passage from non-vascular dentine to vascular dentine in the same tooth, the true law of the development of dentine "by centripetal metamorphosis and calcification of the cells of the pulp," was first definitely enunciated and illustrated from observations made on the development of the teeth of fishes.*

It is interesting to observe in this class the process arrested at each of the well marked stages through which the development of a mammalian tooth passes. In all fishes the first step is the simple production of a soft vascular papilla from the free surface of the buccal membrane: in sharks and rays these papillæ do not proceed to sink into the substance of the gum, but are covered by caps of an opposite free fold of the buccal membrane: these caps do not contract any organic connection with the papilliform matrix, but, as this is converted into dental tissue, the tooth is gradually withdrawn from the extraneous protecting cup, to take its place and assume the erect position on the margin of the jaw, (article Pisces, vol. iii, page 976.) Here, therefore, is represented the first and transitory "papillary" stage of dental development in mammals; and the simple crescentic cartilaginous maxillary plate, with the open groove behind containing the germinal papillæ of the teeth, offers in the shark a magnified representation of the earliest condition of the jaws and teeth in the human embryo.

In many fishes, e. g. *lophius esox,* the dental papillæ become buried in the membrane from which they rise, and the surface

* In my Hunterian Lectures, delivered at the Royal College of Surgeons, May, 1839. See also, Compte Rendu de l'Académie des Sciences, Dec. 1839, p. 784; and Odontography, Introduction and part i, *passim.*

to which their basis is attached becomes the bottom of a closed sac: but this sac does not become inclosed in the substance of the jaw; so that teeth at different stages of growth are brought away with the thick and soft gum, when it is stripped from the jaw-bone. The final fixation of teeth, so formed, is effected by the development of ligamentous fibres in the sub-mucous tissue between the jaw and the base of the tooth, which fibres become the medium of connection between those parts, either as elastic ligaments, or by continuous ossification. Here, therefore, is represented the "follicular" stage of the development of a mammalian tooth; but the "eruptive" stage takes place without previous inclosure of the follicle and matrix in the substance of the jaw-bone.

In balistes, scarus, sphyræna, the sparoids, and many other fishes, the formation of the teeth presents all the usual stages which have been observed to succeed each other in the dentition of the higher *vertebrata:* the papilla sinks into a follicle, becomes surrounded by a capsule, and is then included within a closed alveolus of the growing jaw, where the development of the tooth takes place and is followed by the usual eruptive stages. A distinct enamel-pulp is developed from the inner surface of the capsule in balistes, scarus, sargus, and chrysophrys.

The most formidable dentition exhibited in the order of osseous fishes, is that which characterises the sphyræna, and some extinct fishes allied to this predatory genus. In the great barracuda of the southern shores of the United States, (sphyræna barracuda, Cuv.,) the lower jaw contains a single row of large, compressed, conical, sharp-pointed, and sharp-edged teeth, resembling the blades of lancets, but stronger at the base. The two anterior of these teeth are twice as long as the rest, but the posterior and serial teeth gradually increase in size towards the back part of the jaw; there are about twenty-four of these piercing and cutting teeth in each premandibular bone. They are opposed to a double row of similar teeth in the upper jaw, and fit into the interspace of these two rows when the mouth is closed. The outermost row is situated on the intermaxillary, the innermost on the palatine bones; there are no teeth on the vomer or superior maxillary bones. The two anterior teeth in each premaxillary bone, equal the opposite pair in the lower jaw in size: the posterior teeth are serial, numerous and small size; the second of the two anterior large premaxillary teeth is placed on the inner side of the commencement of the row of small teeth, and is a little inclined backwards. The retaining power of all the large anterior teeth is increased by a slight posterior projection, similar to the barb of a fish hook, but smaller. The palatine bones contain each nine or ten lancet-shaped teeth, somewhat larger than the posterior ones of the lower jaw. All these teeth afford good examples of the mode of attachment by implantation in sockets, which has been denied to exist in fishes.

The loss or injury to which these destructive weapons are liable in the conflict which the sphyræna wages with its living and struggling prey is repaired by an uninterrupted succession of new pulps and teeth. The existence of these is indicated by the foramina, which are situated immediately posterior to, or on the inner margin of, the sockets of the teeth in place; these foramina lead to alveoli of reserve, in which the crowns of the new teeth in different stages of development are loosely imbedded. It is in this position of the germs of the teeth, that the sphyrænoid fishes, both recent and fossil, mainly differ, as to their dental characters, from the rest of the scomberoid family, and proportionally approach the sauroid type. The base or fang of the fully-developed tooth of the sphyræna, is anchylosed to the parietes of the socket in which it is inserted. The pressure of the crown of the new tooth excites absorption of the inner side of the base of the old, which thus finally loses the requisite strength of attachment; and its loss is followed by the absorption of the old socket, as in the higher animals.

It is interesting to observe that the alternate teeth are, in general, contemporaneously shed; so that the maxillary armor is always preserved in an effective state. The relative position of the new teeth to their predecessors, and their influence upon them, resembles, in the *sphyrena*, some of the phenomena which will be described in the dentition of the crocodilian reptiles. To the crocodiles the present voracious fish also approximates in the alveolar lodgment of the teeth; but it manifests its ichyic character in the anchylosis of the fully-developed teeth to their sockets, and still more strikingly in the intimate structure of the teeth.

In all fishes the teeth are shed and renewed, not once only, as in mammals, but frequently, during the whole course of their lives. The maxillary dental plates of *lepidosiren*, the cylindrical dental masses of the chimæroid and edaphodont fishes, and the rostral teeth of *pristis*, if these modified dermal spines may be so called, are, perhaps, the sole examples of "permanent teeth" to be met with in the whole class.

When the teeth are developed in alveolar cavities, they are usually succeeded by others in the vertical direction, as in the pharyngeal bones of the labroids (article Pisces, vol. iii. page 978;) but sometimes they follow one after the other, side by side, as in the scaroids (Pisces, page 979.) The successional teeth owe the origin of their matrix to the budding out from the capsule of their predecessors of a cæcal process, in which the papillary rudiment of the dentinal pulp is developed according to the laws explained in the introduction to my "Odontography," and the article TOOTH. But, in the great majority of fishes, the germs of the new teeth are developed, like those of the old, from the free surface of the buccal membrane throughout the entire period of succession; a circumstance peculiar to the present class. The angler, the pike, and most of our common fishes, illustrate this mode of dental reproduction; it is very conspicuous in the cartilaginous fishes, in which the whole phalanx of their numerous teeth is ever marching slowly forwards in rotatory progress over the alveolar border of the jaw, the teeth being successively cast off as they reach the outer margin, and new teeth rising from the mucous membrane behind the rear rank of the phalanx.

This endless succession and decadence of the teeth, together with the vast numbers in which they often coexist in the same fish, illustrate the law of vegetative or irrelative repetition, as it manifests itself on the first introduction of new organs in the animal kingdom, under which light we must view the above described organized and calcified preparatory instruments of digestion in the lowest class of the vertebrate series.

At the extreme limit of the class of fishes, and connecting that class with the reptiles, stands the very remarkable genus, the dental system of which is figured in cut 17. This consists of two small, slender, conical, sharp-pointed, and slightly recurved teeth, which project downwards from the nasal bone (*c*,) and of strong, trenchant dental plates anchylosed with the alveolar border of the upper (*a*) and lower (*b*) jaws, in each of which the plate is divided at the middle, or symphysial line, so as to form two distinct lateral teeth.

The office of the two laniariform teeth is to pierce and retain the nutritive substance or prey which is afterwards divided and comminuted by the strong maxillary dental plates.

The upper pair of these plates is supported by the anterior part of a strong arch of bone, which combines the characters of the superior maxillary, palatine, and pterygoid bones; the superior maxillary is represented by the median and anterior bar, passing in front of the dental plate of the lower jaw when the mouth is shut, terminating on each side in a process which projects outwards and backwards, as in fig. 17, (*a*,) on each side of the anterior part of the arch; the palatine portion constitutes the median part of the roof of the mouth behind the foregoing; the pterygoid portion is indicated by its fulfilling the usual function of an abutment extended between the palatine portion of the upper jaw and the articular pedicle of the lower jaw; the upper dental plates are confined to the first two parts of the arch, maxillary and palatine, and do not extend upon the pterygoid portion; the lower dental plates (*b*) are anchylosed to the premandibular bone. Viewing the upper pair of plates as a single tooth, it may be described as indented at its outer surface by five vertical angular notches, penetrating inwards through half the breadth of the supporting bone, and dividing the plate into six angular processes, which from the direction and varying form and breadth of the entering notches, radiate from the posterior part of the median line or division of the tooth. The inferior dental plate is similarly notched on its outer side, but the proportions of the angular indentations are such that they receive all the processes of the upper dental plate when the mouth is shut, whilst only the four anterior processes are reciprocally received into the notches of the upper dental plate, this, with the supporting arch, being anterior to the lower plate—a position which is decisive in favor of its maxillary character, and against its homology with the vomer.

The dental plate consists, as in the cod and *sphyræna*, of a central mass of coarse osseous substance, traversed by large and nearly parallel medullary canals, and an external sheath of very hard "vitro-dentine." The medullary canals are continued from a coarse reticulation of similar but wider canals in the substance of the supporting bone, and advance forwards, nearly parallel with each other and with the plane of the upper surface of the tooth; they anastomose together by short, curved, transverse canals, which intercept spaces increasing in length as the canals recede from the osseous basis. The canals, themselves, diminish in size in the same ratio; and when they have arrived near the dense outer layer, their divisions and inosculations become again more frequent, the peripheral loops forming a well-marked line of demarcation between the coarse tubed and the fine tubed dentine. The interspaces of the medullary canals are occupied by a clear substance, and by moss-like reticulations of fine dental tubes, which appear to be more sparing in number than in the teeth of the *sphyræna* or shark. The dentinal tubes of the vitro-dentine run nearly parallel to each other, and vertically to the external surface of the dental plate through about two-thirds of the thickness of that tissue; they then bend and cross each other in a manner very similar to those of the vitro-dentine in the teeth of the *lepidotus*, *phyllodus*, &c.

In the process of attrition this external dense substance is worn away from the upper surface of the dental processes in the lower jaw, exposing the softer vaso-dentinal substance of the tooth; in this state, the dental plate offers an analogy to the incisors of the rodents, a posterior softer substance being sheathed by an anterior denser layer; and an external sharp edge is similarly kept up by the unequal wearing away of the two substances. The progressive waste at the upper surface of the dental plate, would appear to be met by a corresponding addition of new material to its lower part.

In the structure here presented, we have a condition of the dentine which has hitherto been met with only in the class of fishes.

The test of the affinities of the present paradoxical genus, afforded by the microscopic examination of the teeth, gives additional confirmation to the view to which I have been led, from arguments drawn from the rest of its organization, that the *lepidosiren* is in every essential point, a member of the class of fishes.*

### *Dental System of Reptiles.*

If we compare the dental system of the foregoing batrachoid fish with that in the true *batrachia*, it is only to the larval state of the anourans that an analogy can be found; the tadpole of the frog having its maxilla and mandibula each sheathed with a single and continuous horny dental trenchant covering. Were this sheath actually dentinal in tissue and united to the jaw-bone, the resemblance to the *lepidosiren* would be closer; but in point of fact the analogy is very remote; the horny beak of the tadpole is never calcified or anchylosed, but is shed during the progress of the metamorphosis.† The siren alone, among

*Linnean Transactions, vol. xviii, 1839, p. 350. That the large size, or elliptical form of its blood-discs should outweigh the cumulative evidence establishing the piscine nature of the *lepidosiren* could only be surmised by those who are ignorant of the variation in size and shape which the blood-discs present in the class of fishes, and the consequent unimportance of those particles as a character of the class. As well might the *petromyzon* be deemed a mammal because its blood-discs are circular and comparatively small, as the *lepidosiren* be held to be a batrachian because its blood-discs are elliptical and comparatively large.

†The large dental plates of *lepidosiren* have their nearest homologues in those of the extinct fish called *ceratodus* (Odontography, pl. 22, fig. 2.)

the larval-like perennibranchiate reptiles, retains the sheath upon the extremity of the upper and lower jaws; it consists of a firm albuminous tissue, and becomes harder than horn. But these trenchant mandibles, which play upon one another like the blades of a pair of curved scissors, are associated with numerous small but distinct true teeth, which are grouped together to form a rasp-like surface on each half of the divided vomer, and which beset the alveolar border of the splenial element of the mandible below.

In the class *reptilia*, the whole order of *chelonia* is edentulous, as well as the whole family of toads (bufonidæ) in the order *batrachia;* certain extinct genera of saurians were likewise edentulous, *e. g.* the remarkable "*rhynchosaurus*" of the new red sandstone of Shropshire, and some of the extinct saurians of South Africa.

In the tortoises and turtles the jaws are covered by a sheath of horn, which in some species is of considerable thickness and very dense; its working surface is trenchant in the carniverous species, but variously sculptured, and adapted for both cutting and bruising in the vegetable feeders; it may be said that the transitory condition of the mandibles of the batrachian larvæ is here persistent.

The development of the continuous horny maxillary sheath commences, as in the parrot tribe, from a series of distinct papillæ, which sink into alveolar cavities, regularly arranged (in *trionyx*) along the margins of the upper and lower jaw-bones: these alveoli are indicated by the persistence of vascular canals long after the originally separate tooth-like cones have become confluent, and the horny sheath completed.

The teeth of the dentigerous saurian, ophidian, and batrachian reptiles, are, for the most part, simple and adapted for seizing and holding, but not for dividing or masticating their food. The siren alone, combines true teeth with a horny maxillary trenchant sheath, like that of the chelonian reptiles.

With respect to number, in no existing reptile are the teeth reduced so low as in certain mammals and fishes; nor, on the other hand, are they ever so multiplied as in many of the latter class. The extinct dicynodont reptiles of South Africa had but two teeth, which were long tusks implanted in the upper jaw. Some species of amphisbæna, (A. alba,) with fifteen teeth in the upper jaw, and fourteen in the lower jaw, and certain monitors, (varanus,) with sixteen teeth in the upper, and fourteen in the lower jaw, afford examples of the smallest number of teeth amongst existing reptiles; and certain batrachians, with teeth "en cardes" at the roof of the mouth, or which have upwards of eighty teeth in each lateral maxillary series, present the largest number. It is rarely that the number of the teeth is fixed and determinate in any reptile so as to be characteristic of the species, and still more rarely have the individual teeth such characters as to be determined homologically from one species to another.

With respect to situation, the teeth may be present on the jaws only, i. e. the maxillary, premaxillary, and mandibular bones, as in the crocodiles and many lizards: or upon the jaws and roof of the mouth; and here either upon the pterygoid bones, as in the iguana and mosasaur, or upon both palatine and pterygoid bones, as in most serpents, or upon vomer, as in most batrachians, or upon both vomerine and pterygoid bones, as in the axolote, or upon the vomerine and sphenoid bones, as in the salamandra glutinosa, maclure. With respect to the marginal or jaw teeth, these may be absent in the intermaxillary bones, as in many serpents; or they may be present in the upper and not in the lower jaw, as in most frogs; or in both upper and lower jaws, as in the tailed batrachians; and among these they may be supported, upon the lower jaw, by the premandibular or dentary piece, as in the salamanders, menopome, amphiume, proteus; or upon the splenial piece, as in the siren; or upon both splenial and premandibular bones, as in the axolote. The palatine and pterygoid teeth may, in the batrachians, be arranged in several rows, like the "dents en cardes" of fishes. The sphenoid and splenial teeth are always so arranged in the few species that possess them. The intermaxillary, maxillary, and premandibular teeth are uniserial, or in one row, with the exception of the cæcilia and the extinct labyrinthodonts, which have a double row of teeth at the anterior part of the lower jaw.

The teeth of reptiles, with few exceptions, present a simple conical form, with the crown more or less curved, and the apex more or less acute. The cone varies in length and thickness; its transverse section is sometimes circular, but more commonly elliptical or oval, and this modification of the cone may be traced through every gradation, from the thick, round, canine-like tooth of the crocodile, to the sabre-shaped fang of the varanus, the megalosaur, and the cladeiodon. Sometimes as in the fully formed teeth of the megalosaur, one of the margins of the compressed crown of the tooth is trenchant, sometimes both are so; and these may be simply sharp-edged, as in the varanus of timor, or finely serrated, as in the great varanus, the cladeiodon, and the megalosaur.

The outer surface of the crown of the tooth is usually smooth; it may be polished, as in the leiodon, or impressed with fine lines, as in the labyrinthodon, or raised into many narrow ridges, as in the pleiosaur and polyptychodon, or broken by a few broad ridges, as in the iguanodon, (fig. 28,) or grooved by a single longitudinal furrow, as in some serpents, (fig. 26, *a.)*

The cone is longest, and its summit sharpest, in the serpents; from these may be traced, chiefly in the lizard tribe, a progressive shortening, expansion of the base, and blunting of the apex of the tooth, until the cone is reduced to a hemispherical tubercle, or plate, as in the thorictes and cyclodus, (fig. 27.)

In the pleiosaur the dental cone is three-sided, with one of the angles rounded off. The posterior sub-compressed teeth of the alligator, (fig. 30,) present a new modification of form; here they terminate in a mammillate summit, supported by a slightly constricted neck. In the tooth of the hylœosaur, the expanded summit is flattened, bent, and spear-shaped, with the edges blunted. But the expansion of the crown is greatest in the sub-compressed teeth of the extinct cardiodon and the existing iguanas, the teeth of which are farther complicated by having the margins notched. The great iguanodon had the crown of the tooth expanded, both in length and breadth, and combining marginal dentations with longitudinal ridges: this tooth, (fig. 28,) presents the most complicated external form as yet discovered in the class of reptiles.

In no reptiles does the base of the tooth ever branch into fangs.

*Attachment.*—As a general rule, the teeth of reptiles are anchylosed to the bone which supports them. When they continue distinct, they may be lodged either in a continuous groove, as in the ichthyosaur, or in separate sockets, as in the plesiosaur and crocodilians (fig. 30.) The base of the tooth is anchylosed to the walls of a moderately deep socket in the extinct megalosaur and theocodon. In the labyrinthodonts and cæciliæ, among the bratrachians; in most ophidians; and in the geckos, agamians, and varanians, among the saurians, the base of the tooth is imbedded in a shallow socket, and is confluent therewith.

In the scincoidians, the safeguards, (tejus,) in most iguanians, in the chameleons and most other lacertian reptiles, the tooth is anchylosed by an oblique surface extending from the base more or less upon the outer side of the crown to an external alveolar plate of bone, the inner alveolar plate not being developed. In the frogs, the teeth are similarly, but less firmly attached to an external parapet of bone. The lizards, which have their teeth thus attached to the side of the jaw are termed pleurodonts. In a few iguanians, as the istiures, the teeth appear to be soldered to the margins of the jaws, these have been termed "acrodonts." In some large extinct lacertians, *e. g.* the mosasaur and leiodon, the tooth is fixed upon a raised conical process of bone. These modifications of the attachment of the teeth of reptiles are closely adapted to the destined application of those instruments, and relate to the habits and food of the species; we may likewise perceive that they offer a close analogy to some of the transitory conditions of the human teeth. There is a period, for example, when the primitive dental papillæ are not defended by either an outer or an inner alveolar process, any more than their calcified homologues which are confluent with the margin of the jaw in the rhynchocephalus. There is another stage in which the groove containing the dental germs is defended by a single external cartilaginous alveolar ridge; this condition is permanetly typified in the cyclodus, (fig. 27, and most existing lizards. Next there is developed in the human embryo an internal alveolar plate, and the sacs and pulps of the teeth sink into a deep, but continuous groove, in which traces of transverse partitions soon make their appearance; in the ancient ichthyosaur the relation of the jaws to the teeth never advanced beyond this stage.

Finally, the dental groove is divided by complete partitions, and a separate socket is formed for each tooth; and this stage of development is attained in the highest organized reptiles, *e. g.* the crocodiles (fig. 30.)

*Substance.*—This may be four-fold, and a single tooth may be composed of dentine, cement, enamel, and bone; but the dentine and cement are present in the teeth of all reptiles.

In the batrachians and ophidians, a thin layer of cement invests the central body of dentine, and, as usual, follows any inflections or sinuosities that may characterise the dentine. Besides the outer coat of cement, which is thickest at the base of the teeth, a generally thin coat of enamel defends the crown of the tooth in most saurians, and the last remains of the pulp are not unfrequently converted into a coarse bone, both in the teeth which are anchylosed to the jaw, and in some teeth, as those of the ichthyosaur, which remain free. The only modification of the dentine, which could at all entitle it to be regarded in the light of a new or distinct substance, is that which is peculiar in the present class to the teeth of the iguanodon, and which will be described in the following section.

*Structure.*—The varieties of dental structure are few in the reptiles as compared with either fishes or mammals, and its most complicated condition arises from interblending of the dentinal and other substances, rather than from modifications of the tissues themselves. In the teeth of most reptiles the intimate structure of the dentine corresponds with that which has been described as the type of the tissue, *e. g.* the hard or unvascular dentine, and which is the prevailing modification in mammalia, viz. the radiation of a system of minute plasmatic tubes from a single pulp-cavity, at right angles to the external surface of the tooth. The most essential modification of this structure is the intermingling of cylindrical processes of the pulp-cavity in the form of medullary canals, with the finer tubular structure. Another modification is that in which the dentine maintains its normal structure, but is folded inwardly upon itself, so as to produce a deep longitudinal indentation on one side of the tooth; it is the expansion of the bottom of such a longitudinal deep fold that forms the central canal of the venom-fang of the serpent; but a glance at fig. 25, will show that, notwithstanding the singularly modified disposition of the dentine *(b,)* its structure remains unaltered; and although the pulp-cavity *(p)* is reduced to the form of a crescentic fissure, the dentinal tubes continue to radiate from it according to the usual law. By a similar inflection of many vertical longitudinal folds of the external cement and external surface of the tooth at regular intervals around the entire circumference of the tooth, and by a corresponding extension of radiated processes of the pulp-cavity and dentine into the interspaces of such inflected and converging folds, a modification of dental structure is established in certain extinct reptiles, which, by the various sinuosities of the interblended folds of cement and processes of dentine, with the partial dilatations of the radiated pulp-cavity, produces the complicated structure which is described in fig. 9. But this complication is nevertheless referable to a modification of form or arrangement of the dental tissues, rather than of the structure of the tissues themselves: the calcigerous tubes in each sinuous lobe of dentine, in the most complex tooth of the labyrinthodon, exhibit the same general disposition and course, as in the fang of the serpent and in the still more simple tooth of the saurian.

*Development.*—The teeth of reptiles are never completed, as in certain fishes, at the first or papillary stage; but the pulp sinks into a follicle, and becomes inclosed by a capsule; and in certain reptiles this becomes more or less surrounded by bone ; but the process of development never offers the eruptive stage, in the sense in which this is usually understood, as signifying the extrication of the young tooth from a closed alveolus.

The completion of a tooth, with the extinct exception of the dicynodont reptiles, is soon followed by preparation for its removal and succession: the faculty of developing new tooth-germs seems to be unlimited in the present class, and the phenomena of dental decadence and replacement are manifested at every period of life; the number of teeth is generally the same in each successive series, and the difference of size presented by the teeth of different and distant series is considerable.

The new germ is always developed, in the first instance, at the side of the base of the old tooth, never in the cavity of the base; the crocodiles form no exception to this rule. The poison-fangs of serpents succeed each other from behind forwards; in almost every other instance the germ of the successional tooth is developed at the inner side of the base of its predecessor. In the frog the dental germ makes its appearance in the form of a papilla developed from the bottom and towards the outer side of a small fissure in the mucous membrane or gum that fills up the shallow groove at the inner side of the alveolar parapet and its adherent teeth: the papilla is soon enveloped by a capsular process of the surrounding membrane: there is a small enamel pulp developed from the capsule opposite the apex of the tooth; the deposition of the earthy salts in this mould is accompanied by ossification of the capsule, which afterwards proceeds *pari passu* with the calcification of the dentinal papilla or pulp; so that, with the exception of its base, the surface of the uncalcified part of the pulp alone remains normally unadherent to the capsule.

As the tooth acquires hardness and size, it presses against the base of the contiguous attached tooth, causes a progressive absorption of that part, and finally undermines, displaces and replaces its predecessor. The number of nascent matrices of the successional teeth is so great in the frog, and they are crowded so close together, that it is not unusual to find the capsules of contiguous tooth-germs becoming adherent together, as their ossification proceeds. After a brief maceration, the soft gum may be stripped from the shallow alveolar depression, and the younger tooth-germs in different stages of growth are brought away with it.

The mode of development of the teeth of serpents does not differ essentially from that of the teeth of the batrachian above described, except in the relation of the papillæ of the successional poison-fangs to the branch of the poison-duct that traverses the cavity of the loose mucous gum in which they are developed.

*Batrachian modifications.*—Some of the peculiarities of the dentition of the batrachians have already been noticed, as in the comparison of the *siren* with the *lepidosiren,* in which the true amphibian was shown to have numerous teeth on the palate and lower jaw.

The piscine character of rasp-like teeth aggregated in numerous series, is manifested also in the axolotl, upon the palatal region of the mouth, and upon the splenial or opercular element of the lower jaw; but the superior maxillary bones are here developed, and also support teeth. The premandibular and the premaxillary bones, instead of preserving the larval condition of the horny sheath, have their alveolar border armed with a single row of small, equal, fine and sharp-pointed denticles, which are continued above, along the maxillaries; thus establishing the commencement of the ordinary batrachian condition of the marginal teeth of the buccal cavity. The dentigerous bones of the palate consist of two plates on each side, as in the *siren;* the anterior pair, or vomerine bones, converge and meet at their anterior extremities; the minute denticles which they support are arranged quinceuncially; the posterior pair of bones are continued backwards according to the usual disposition of the pterygoids, to abut against the tympanic bones; the denticles are confined to the anterior part of their oral surface, and resemble in their arrangement and anchylosed attachment those of the vomerine series, of which they form the posterior termination.

The frogs *(rana)* have no teeth on the lower jaw; but in some species the alveolar edge of this bone is finely notched or dentated, as in the horned frogs *(ceratophrys.)* The inter-maxillary and maxillary bones support a long, close-set, single series of small, conical, hollow teeth, of which the apices only project beyond the external alveolar ridge to which they are attached. A short transverse row of similar but smaller teeth extends along the posterior border of each vomer, except in the slender-armed frogs, (*leptobrachium*,) and in some of the tree frogs, (e. g. *euchnemes*,) in which the roof of the mouth is edentulous.

Amongst the most extraordinary examples of extinct reptiles, are those which are characterised by the labyrinthic modifications of the dental structure above described, and which with some affinities to saurians, combine characters which are essentially those of the order *batrachia.* I have ascertained by fossil portions of the upper jaw of the *labyrinthodon leptognathus*, that the maxillary or facial division of the skull was broad, much depressed, and flattened, resembling the skull of the gigantic salamander and of the alligator; and the outer surface of the bones was strongly sculptured, as in the crocodilian family, but of a relatively larger and coarser pattern. The upper jaw contains a single row of small teeth, about sixty in number, anterior to which are three or four large conical tusks. The bases of the serial teeth project directly from the outer wall of the shallow socket, there being no alveolar ridge external to it. The second large anterior tusk is three times the size of the first of the serial teeth, and the size of these teeth gradually diminishes as they are placed further back; the length of the common-sized teeth being about two lines, and the greatest breadth one-third of a line. The apical two-thirds of each tooth is smooth, but the basal third is fluted and anchylosed to the outer wall of the socket. The osseous roof of the mouth is principally composed of a pair of broad and flat bones, homologous with the divided vomer in *batrachia*, but of much greater relative extent, approaching in this respect, those of the menopome, and defending the mouth with a more extensive roof of bone than exists in any lacertian reptile; physiologically, therefore, the *labyrinthodon*, in this part of its structure, comes nearest to the crocodile; but the structure itself, morphologically, is essentially batrachian. In the menopome and gigantic salamander, a row of small teeth extends transversely across the anterior extremity of the vomerine bones; and the occurrence in the *labyrinthodon* of a similar row, consisting in each palatine bone of three median small teeth and two outer large ones, marks most strongly its batrachian nature; and from the outermost tooth, a longitudinal row of small and equal-sized teeth is continued backward along the exterior margin of the palatine bone. The whole of this series of palatal teeth is nearly concentric with the maxillary teeth.

In lacertine reptiles the examples of a row of palatal teeth are rare, and, when present, it is short, and situated towards the back of the palate, upon the pterygoid bones, as in the iguana and mosasaur. In batrachians the most common disposition of the palatal teeth is a transverse row placed at the anterior part of the divided vomer, as in frogs, the menopome, and gigantic salamander, and at the posterior part in certain toads. In the amphiume, on the contrary, the palatal teeth form a nearly longitudinal series along the outer margin of the palatine bones. The labyrinthodon combines both these dispositions of the palatal teeth. The lower jaw, like the upper, contains a series of small teeth, with a few larger tusks anterior to them, the serial teeth are long and slender, gradually diminishing in size towards the anterior portion of the jaw; the largest fossil portion which I have obtained presents a linear series of not less than fifty sockets, placed alternately, one nearer the inner, the next nearer the outer side of the jaw. The sockets of the teeth are shallower than in the upper jaw; the outer wall is more developed than the inner, and the anchylosed bases of the teeth more nearly resemble, in their oblique position, those of existing *batrachia*. With regard to the modification of the microscopic structure of the teeth, I may observe that, between the apex and the part where the inflected vertical folds of the cement commence, the tooth resembles, in the simplicity of its intimate structure, that of the entire tooth of the ordinary *batrachia* and most reptiles; and in the lower or basal half of the tooth the labyrinthic structure above described commences, and gradually increases in complexity.

In the genus *deirodon*,* the teeth of the ordinary bones of the mouth are so small as to be scarcely perceptible; and they appear to be soon lost, so that it has been described as edentulous, and has been called "anodon." An acquaintance with the habits and food of this species has shown how admirably this apparent defect is adapted to its well-being. Its business is to restrain the undue increase of the smaller birds by devouring their eggs. Now if the teeth had existed of the ordinary form and proportions in the maxillary and palatal regions, the egg would have been broken as soon as it was seized, and much of the nutritious contents would have escaped from the lipless mouth of the snake in the act of deglutition; but, owing to the almost edentulous state of the jaws, the egg glides along the expanded opening unbroken; and it is not until it has reached the gullet, and the closed mouth prevents any escape of the nutritious matter, that the egg becomes exposed to instruments adapted for its perforation. These instruments consist of the inferior spinous processes (hypapophyses) of the seven or eight posterior cervical vertebræ, the extremities of which are capped by a layer of hard cement, and penetrate the dorsal parietes of the œsophagus. They may be readily seen, even in very small subjects in the interior of that tube, in which their points are directed backwards. The shell being sawed open longitudinally by these vertebral teeth, the egg is crushed by the contractions of the gullet, and is carried to the stomach, where the shell is no doubt soon dissolved by the acid gastric juice.

In the *boa constrictor*, the teeth are slender, conical, suddenly bent backwards and inwards above their base of attachment; the crown is straight or very slightly curved, *e. g.* in the posterior teeth. The intermaxillary bone supports four small teeth; each maxillary bone has eight much larger ones, which gradually decrease in size as they are placed further

* The *coluber scaber* of Linnæus; an arboreal serpent of South Africa.

back. There are eight or nine teeth of similar size and proportions in each premandibular bone. These teeth are separated by wide intervals, from which other teeth, similar to those in place, have been detached. The base of each of the above teeth is extended transversely, compressed antero-posteriorly, and anchylosed to a shallow alveolus, extending obliquely across the shallower alveolar groove. An affinity to the lizard tribes is manifested by the greater development of the outer, as compared with the inner wall of the alveolar furrow.

The palatine teeth, of which there are three or four in each palatal bone, are as large as the superior maxillary, and are similarly attached. The pterygoid teeth, five or six in number, which complete the internal dental series on the roof of the mouth, are of smaller size, and gradually diminish as they recede backwards. In the interspaces of the fixed teeth in both these bones, the places of attachment of the shed teeth are always visible; so that the dental formula, if it included the vacated with the occupied sockets, would express a greater number of teeth than are ever in place and use at the same time. In the smaller species of boa, the intermaxillary bone is edentulous.

The *colubers*, like other true serpents, have two longitudinal rows of teeth on the roof of the mouth, extending along the palatines and pterygoids. The genus *oligodon* appears to form the sole exception to this rule. In the *dryinus nasutus*, a few small teeth are present on the ecto-pterygoid as well as on the pterygoid.

In certain genera of non-poisonous serpents, as *dryophis*, *dipsas* and *bucephalus*, in which the superior maxillary teeth increase in size towards the posterior part of the bone, the large terminal teeth of the series are traversed along their anterior and convex side by a longitudinal groove. In the *bucephalus capensis*, the two or three posterior maxillary teeth present this structure, and are much larger than the anterior teeth, or those of the palatine and premandibular series. They add materially, therefore, to the power of retaining the prey, and may conduct into the wounds which they inflict an acrid saliva; but they are not in connection with the duct of an express poison-gland. The long-grooved fangs are either firmly fixed to the maxillary bones, or are slightly movable, according to their period of growth. They are concealed by a sheath of thick and soft gum, and their points are directed backwards. The sheath always contains loose recumbent grooved teeth, ready to succeed those in place.

In most of the *colubri*, each maxillary and premandibular bone includes from twenty to twenty-five teeth. They are less numerous in the genera *tortrix* and *homalopsis*, and are reduced to a still smaller number in the poisonous serpents, in the typical genera of which the short maxillary bone supports only a single perforated fang.

*Poisonous Serpents.*—The transitions to these serpents, which was begun in the bucephali and allied genera with grooved maxillary teeth, is completed by the poisonous serpents of the genera pelamis, hydrophis, elaps, bongarus, and hamadryas.

The superior maxillary bone diminishes in length with the decreasing number of teeth which it supports. The ecto-pterygoid bone elongates in the same ratio, so as to retain its position as an abutment against the shortened maxillary, and the muscles implanted into this external pterygoid bone communicate through it to the maxillary bone the hinge-like movements backwards and forwards upon the ginglymoid articulations connecting that bone with the prefrontal and palatine bones. As the fully developed poison-fangs are attached by the same firm basal anchylosis to maxillary sockets, which forms the characteristic mode of attachment of the simple or solid teeth, they necessarily follow all the movements of the superior maxillary bone. When the external pterygoid is retracted, the superior maxillary rotates backwards, and the poison-fang is concealed in the lax mucous gum, with its point turned backwards. When the muscles draw forward the external pterygoid, the superior maxillary bone is pushed forwards, and the recumbent fang withdrawn from its concealment and erected.

In this power of changing the direction of a large tooth, so that it may not impede the passage of food through the mouth, we may perceive an analogy between the viper and the lophius; but in the fish, the movement is confined to the tooth alone, and is dependent on the mere physical property of the elastic medium of attachment; in the serpent, the tooth has no independent motion, but rotates with the jaw, whose movements are governed by muscular actions. In the fish, the great teeth are erect, except when pressed down by some extraneous force. In the serpent, the habitual position of the fang is the recumbent one, and its erection takes place only when the envenomed blow is to be struck.

A true idea of the structure of a poison-fang will be formed by supposing the crown of a simple tooth, as that of a boa, to be pressed flat, and its edges to be then bent towards each other, and soldered together so as to form a hollow cylinder, or rather cone, open at both ends. The flattening of the fang, and its inflection around the poison-duct commences immediately above the base, and the suture of the inflected margins runs along the anterior and convex side of the recurved fang, as shown in fig. 24, *a;* the poison canal is thus in front of the pulp-cavity, as shown in the longitudinal section of the fang *b*. The basal aperture of the poison-canal *v*, is oblique, and its opposite outlet *v'*, is still more so, presenting the form of a narrow elliptical longitudinal fissure terminating at a short distance from the apex of the fang. The relative position of the two apertures of the poison-canal is shown in the figure of the fang of the large cobra in my "Odontography," vol. iv. p. 290, art. Reptilia, where a fine hair is represented as passing through the poison-canal.

The poison-glands occupy the sides of the posterior half of the head; each gland consists of a number of elongated narrow lobes, extending from the main duct, which runs along the lower border of the gland upwards, and slightly backwards; each lobe gives off lobules throughout its extent, thus presenting a pinnatifid structure; and each lobule is subdivided into smaller secerning cæca, which constitute the ultimate structure of the gland. The whole gland is surrounded by a double aponeurotic capsule, of which the outermost and strongest layer is in connection with the muscles by whose contraction the several cæca and lobes of the gland are compressed and emptied of their secretion. This is then conveyed by the duct to the basal aperture of the poison-canal of the fang. We may suppose, that as the analogous lachrymal and salivary glands in other animals are most active during particular emotions, so the rage which stimulates the venom-snake to use its deadly weapon must be accompanied with an increased secretion and great distension of the poison-glands; and as the action of the compressing muscles is contemporaneous with the blow by which the serpent inflicts the wound, the poison is at the same moment injected with force into the wound from the apical outlet of the perforated fang.

The duct which conveys the poison, although it runs through the center of a great part of the tooth, is really on the outside of the tooth, the canal in which it is lodged and protected, being formed by a longitudinal inflection of the dentinal parietes

Fig. 24.

*Poison-fangs of Serpents. (Magnified.)*

of the pulp-cavity. This inflection commences a little beyond the base of the tooth, where its nature is readily appreciated, as the poison-duct there rests in a slight groove or longitudinal indentation on the convex side of the fang; as it proceeds it sinks deeper into the substance of the tooth, and the sides of the groove meet and seem to coalesce, so that the trace of the inflected fold ceases, in some species, to be perceptible to the naked eye; and the fang appears, as it is commonly described, to be perforated by the duct of the poison-gland. In the hydrophis the groove remains permanently open, as in fig. 24, *c*.

From the real nature of the poison-canal it follows that the transverse section of the tooth varies in form in different parts of the tooth; at the base it is oblong, with a large pulp-cavity of a corresponding form, with an entering notch at the anterior surface; farther on the transverse section presents the form of a horse-shoe, and the pulp-cavity that of a crescent, the horns of which extend into the sides of the deep cavity of the poisoned-fang; a little beyond this part, the section of the tooth itself is crescentic, with the horns obtuse and in contact, so as to circumscribe the poison-canal; and along the whole of the

Fig. 25.

*Section of poison-fang of Serpent. (Magnified.)*

middle four-sixths of the tooth, the section, of which a magnified view is given in fig. 25, shows the dentine of the fang inclosing the poison-canal, and having its own center or pulp-canal *p*, *p*, in the form of a crescentic fissure, situated close to the concave border of the inflected surface of the tooth. The pulp-cavity disappears, and the poison-canal again resumes the form of a groove near the apex of the fang, and terminates on the anterior surface in an elongated fissure.

The venom-fangs of the viper, rattle-snake, and the fer-de-lance are coated only with a thin layer of a sub-transparent and minutely cellular cement. The disposition of the dentinal tubes is obedient to the general law of verticality to the external surface of the tooth; it is represented as seen in the transverse section from the middle of the fang in fig. 25. Since the inflected surface of the tooth can be exposed to no other pressure than that of the turgescent duct with which it is in contact, the tubes which proceed to the surface *d*, while maintaining their normal relation of the right angle to it, are extremely short; and the layer of dentine separating the poison-tube from the pulp-cavity is proportionally thin. The calcigerous tubes that radiate from the opposite side of the pulp-cavity to the exposed surface *b* of the tooth are disproportionally long.

The teeth of ophidians are developed and completed in that part which forms the original seat of the tooth-germs in all animals; viz. the mucous membrane or gum covering, the alveolar border of the dentigerous bones. This germ presents the same lax tissue, and is as abundantly developed, as in the pike, lophius, and many other fishes; in which it likewise serves as the nidus and locality for the complete development of the teeth. The primitive dental papillæ in the common harmless snake very soon sinks into the substance of the gum, and becomes inclosed by a capsule. As soon as the deposition of the calcareous salts commences in the apex of the papilla the capsule covering that part becomes ossified and adherent to the dentine, and the tooth begins to pierce and emerge from the gum before its mould, the pulp, is half completed. Fresh layers of cells are successively added to the base of the pulp, and converted, by their confluence and calcification, into the tubular dentine, until the full size of the tooth is attained, when its situation in the gum is gradually changed, and its base becomes anchylosed to the shallow cavity of the alveolar surface of the bone.

In the posterior part of the large mucous sheath of the poison-fang, the successors of this tooth are always to be found in different stages of development; the pulp is at first a simple papilla, and when it has sunk into the gum the succeeding portion presents a depression along its inferior surface, as it lies horizontally, with the apex directed backwards; the capsule adheres to this inflected surface of the pulp; and the base of the groove of the loose, growing, poison-fang is brought into the same relation with the duct of the poison gland as the displaced fang, which has been severed from the duct.

*Saurians.*—The existing species of lizards differ from those of the crocodile in the anchylosed condition of the teeth, which present few modifications of importance; those that yield most fruit to physiology, and which have most expanded our ideas of the extent of the resources of nature, and the exceptional deviations from what was deemed the rule of structure in the saurian dentition, have been discovered by the study of the fossil teeth of extinct forms of the order. Amongst these the most extraordinary in respect of their dental system have been recently discovered in a formation in South Africa, which seems nearly as ancient as our own coal-seams. I have called them "dicynodonts"* from their detention being reduced to one long and large canine tooth on each side of the upper jaw. As these teeth give, at first sight, a character to the jaws like that which the long poison-fangs give, when erected, to the jaws of the rattle-snake, I shall briefly notice their characters before entering upon the description of the more normal saurian dentition.

Fig. 26, gives a reduced side view of the skull of the species of dicynodon called D. lacerticeps. The cranial cavity, (8, 8,) is extremely contracted, as in all the cold-blooded

* From δὶς, two, and χυνοδους, the name given by Hippocrates to the canine teeth, and signify the same idea as their common English denomination.

quadrupeds; it is bounded on each side by wide and deep temporal fossæ (*t*) indicating powerful muscles for the action

Fig. 26.

*Skull of Dicynodon lacerticeps, one-third natural size.*

of the lower jaw. The orbits (*o*) are large and round; the nostrils (*n*) are divided by the junction of the nasal bones (15) with the premaxillaries (22) as in lizards; there is not a single median external nostril, as in chelonian and crocodilian reptiles. The alveolar border of the lower jaw and of the premaxillary part of the upper jaw is trenchant, and seems to have been sheathed with horn.

The maxillary bone (21) is excavated by a wide and deep alveolus, with a circular area of half an inch, and lodges a long and strong, slightly curved, and sharp-pointed, canine tooth or tusk, which projects about two-thirds of its length from the open extremity of the socket. The direction of the tusks is forwards, downwards, and very slightly inwards; the two converging, as they descend along the outer side of the compressed symphysis of the lower jaw (*c*.) The tusk is principally composed of a body of compact unvascular dentine. The base is excavated by a wide conical pulp-cavity (*p*) with the apex extending to about one-half of the implanted part of the tusk, and a linear tract is continued along the center of the solid part of the tusk. From this central line the dentinal tubes radiate, with a gentle curve at the beginning, convex towards the point of the tusk, and then proceeding straight to the periphery of the tooth, but inclining towards the apex. They present parallel secondary curves, divide dichotomously twice or thrice near their beginning and send off numerous small lateral branches chiefly from the side next the apex. At their primary curve the dentinal tubes are $\frac{1}{12000}$ of an inch in diameter, and their intervals are $\frac{1}{6000}$ of an inch across. The dental cells are most conspicuous near the periphery of the tooth, and vary in diameter from $\frac{3}{1000}$ to $\frac{1}{1000}$ of an inch.

The enamel at least at the middle of the tusk, is thinner than in the teeth of the crocodile. It presents only a finely lamellated texture, the layers being parallel with the surface of the dentine on which it rests. There is only a fine linear trace of cement on the exterior of the sections of the implanted base of the tusks ; and here it is too thin to allow of the development of the radiated cells in its substance. There is no trace of teeth or their sockets in the lower jaw (25, 23 ;) so much of the alveolar border as is exposed presents a smooth and even edge, which seems to have played like a scissor-blade upon the inner side of the corresponding edentulous border of the upper jaw ; and it is most probable, from the analogies of similarly shaped jaws of existing reptilia, that the forepart of both the upper and under jaws, were sheathed with horn.

Until the discovery of the rhynchosaurus, this edentulous and horn-sheathed condition of the jaws was supposed to be peculiar to the chelonian order among reptiles ; and it is not one of the least interesting features of the dicynodonts of the African sandstones, that they should repeat a chelonian character, hitherto peculiar, amongst lacertians, to the above-cited remarkable extinct edentulous genus of the new red sandstone of Shropshire : but our interest rises almost to astonishment, when in a saurian skull, we find, superadded to the horn-clad mandibles of the tortoise, a pair of tusks, borrowed as it were from the mammalian class, or rather foreshadowing a structure, which, in the actual creation, is peculiar to certain members of the highest organised warm-blooded animals.

In the other reptilia, recent or extinct, which most nearly approach the mammalia in the structure of their teeth, the difference and characteristic of the inferior and cold-blooded class is manifested in the shape, and in the system of shedding and succession, of the teeth : the base of the implanted teeth seldom becomes consolidated, never contracted to a point, as in the fangs of the simple teeth of mammalia, and at all periods of growth one or more germs of teeth are formed within or near the base of the tooth in use, prepared to succeed it, and progressing towards its displacement. The dental armature of the jaws is kept in serviceable order by uninterrupted change and succession ; but the matrix of the individual tooth is soon exhausted, and the life of the tooth itself may be said to be comparatively short.

The dicynodonts not only manifest the higher type of free implantation of the base of the tooth in a deep and complete socket, common to crocodilians, megalosaurs, and thecodonts, but make an additional and much more important step towards the mammalian type of dentition, by maintaining the serviceable state of the tusk by virtue of constant renovation of the substance of one and the same matrix, according to the principle manifested by the long-lived and ever-growing tusks of the walrus, and the scalpriform incisors of the rodentia.

The genera of the typical family of the squamate lacertians are arranged in two sub-families, the chief characteristics of which are derived from the dental system.

In the first group, the teeth are solid, or without any permanent internal cavity, and are very firmly anchylosed by their base to the alveolar groove upon the inner side of the jaw ; so that the extremity of the tooth is slightly directed outwards. The species which present this character are called pleodonts.

In the second group the teeth are excavated, or retain the pulp-cavity, and are less firmly fixed to the jaws, being applied vertically, like piles or buttresses, against the outer alveolar parapet, but not adhering by their base. The group is called cœlodonts.

The monitor lizard of South America is an example of the pleodont group, in which the premaxillary teeth are ten in number. The maxillary teeth vary from ten to fifteen on each side, and increase in size as they are placed farther back; the hindmost teeth are tricuspid in young individuals, and present the form of simple tubercles in the old monitors. The mandibular teeth, fifteen to eighteen in number in each ramus, correspond in size and form with those above. In the cœlodont group, the "swift lizards," (tachydromus,) have the pterygoid bones armed with minute teeth. The teeth on both upper and lower jaws are of larger size, and the hinder ones are tricuspid. The true lizards, (lacerta,) have two kinds of teeth quoad form; the anterior small, conical, aud recurved; the posterior larger, and bi-or tri-cuspid. Some species have also pterygoid teeth; as the common lacerta agilis.

In the gigantic fossil monitor of Maestricht, the teeth combine the pleodont with the acrodont characters.

The true affinities of the mosasaur, which was at least twenty-four feet in length, and the remains of which characterise the chalk-formations, were first determined by Cuvier, who places it in the lacertian group of saurians, between the iguanæ and monitors. Its dentition exhibits in an eminent degree the acrodont character; the teeth being supported on expanded conical bases anchylosed to the summit of the alveolar ridge of the jaw: no existing saurian exactly parallels this mode of attachment of the teeth, either in regard to the breadth of the alveolar border, or in the relative size of the osseous cones to the teeth which they support. A shallow socket is left where the tooth and its supporting base are shed. The form of the teeth is likewise different from that hitherto observed in any existing saurian: the crown is pyramidal, with the outer side nearly plane, or slightly convex, and separated by two sharp ridges from the remaining surface, which forms a half-cone. All the teeth are slightly recurved, and their peripheral surface is smooth. The teeth are implanted upon the premaxillary, maxillary, and premandibulary bones; a series of similarly shaped but much smaller teeth are placed upon the pterygoid bones.

The gradual transition from the simple structure of the compact dentine to the osteo-dentine of the anchylosed base of the tooth was not known to Cuvier; otherwise he could not have supposed that the crown and the base of the tooth of the mosasaurus were formed by vital processes of so dissimilar a nature as to forbid him considering them as parts of one and the same body. Cuvier had originally described the expanded base of the tooth of the mosasaur as the root of the tooth; but afterwards observing that the corresponding base became anchylosed by ossification of the remains of the pulp to the jaw, he conceived it to be incorrect to regard it as a part of a body which he believed to be an inorganic product, and the result of excretion. "The tooth," he observes, in correcting his first account of the mosasaurus, "has no true root, but it adheres strongly to that pulp which has secreted it, and it is further held in connection with it by the remains of the capsule which has furnished the enamel, and which, by becoming ossified also, and uniting itself to the maxillary bone and the ossified pulp, implants or rivets the tooth with additional force."

The necessity under which Cuvier felt himself compelled to regard the crown and the base of the tooth of the mosasaur as two distinct parts, is at once banished by the recognition of the principle, that the processes of calcifiication are essentially the same at every part of a tooth, whether it be free or anchylosed; and that they are modified only as I have shown in my memoir on the formation of the teeth of the shark, according to the density of the part to be produced.

*Scincoid Lizards.*—Most of these smooth-scaled lizards have small mouths and slender sharp teeth, fitted best for insect food; they are usually confined to the upper and lower jaws; but the medicinal scink of ancient pharmacy (scincus officinalis) has four or five small obtuse teeth upon each pterygoid bone. The chief exception to the typical dentition of the present family is made by the large scincoid lizards of Australia, which, on that account, have received the generic name of *cyclodus.*

The dentition of the *cycl. nigroluteus* is exemplified in the lower jaw, fig. 27. In the upper jaw, the single premaxillary

FIG. 27.

*Lower jaw and teeth of Cyclodus Nigroluteus.*

bone has depressions for twelve teeth, of which only the alternate ones, are usually in place ; they are of very small size, with the fang compressed laterally, and the crown antero-posteriorly, so as to resemble a true incisor in form, the summit sloping to an edge from behind forwards, with the middle of the cutting surface a little produced. Each superior maxillary bone has depressions for fourteen teeth ; they quickly increase in size, and exchange their conical for a sub-hemispherical crown, the eighth to the thirteenth inclusive are the largest teeth ; they are set obliquely, and pretty close together. In the lower jaw there are two small incisors, at the anterior part of each premandibular bone correspoding with those of the premaxillary ; these are succeeded by five or six conical teeth, and the rest correspond in size and form with the tuberculate molars of the upper jaw.

All the teeth are attached, after the pleurodont type, by their base and outer margin to shallow depressions on the outer side of the external alveolar parapet.

The germs of the successional teeth, *c*. fig. 27, are developed at the inner side of the base of their predecessors, *a*, which they excavate, undermine, and displace in the usual manner.

*Iguanas.*—Certain genera of this family of lizards, e. g. *istiurus*, *lophyrus*, *colotes*, and *otocryptis*, have the teeth soldered, like those of *mosasaurus*, to the summit of the alveolar ridge, and thence are called "acrodonts :" in all these lizards the maxillary and mandibular teeth may be divided into anterior laniary, and posterior molary teeth. In most of the iguanians the teeth are lodged in a common shallow oblique alveolar groove, and are soldered to excavations on the inner surface of the outer wall of the groove: these are called pleurodonts. Most of them possess pterygoid as well as maxillary teeth: but the following genera, *hyperanodon*, *tropidolepis*, *phrynosoma*, and *callisaurus*, are exceptions.

In the pleurodont iguanians, the teeth never present the true laniary form; and if simply conical, as at the extremes of the maxillary series, the cone is more or less obtuse; but, in general it is expanded, more or less trilobate, or dentated along the margin of the crown.

The *amblyrhynchus*, a genus which is somewhat remarkable for the marine habits of at least one of the species (amblyrhynchus ater,) whose diet is sea-weed,* has the tricuspid structure well developed in the posterior teeth.

The typical genus of the present family of saurians (iguana tuberculata,) is characterised by the crenate or dentated margin of the crown of the maxillary and premandibular teeth, a few of the anterior small ones excepted. The pterygoid teeth are arranged in two or three irregular rows, resembling somewhat the "dents en cordes" of fishes. In the full grown *iguana tuberculata*, there are from forty-seven to forty-nine teeth in both upper and lower jaws. The number is less in young subjects. The double row of pterygoid teeth are in close order on each side.

In the horned iguana (metopoceros cornutus,) there are about fifty-six teeth in the upper and lower jaws, of which the four first are conical and slightly curved. The twelve succeeding teeth are somewhat larger in size, with more compressed and expanded crowns; the rest are triangular, compressed, with dentated margins. The inner surface of the crown of the tooth is simply convex and smooth; the outer surface traversed by a median longitudinal, broad, obtuse ridge. There is a single row of small teeth implanted in each pterygoid bone. No iguanian lizard has teeth on the palatine bones.

*This species, and probably all the known *amblyrhynchi*, or blunt-nosed iguanæ, inhabit the islands in the Galopagos group; their habits have been well elucidated by Mr. Darwin. (Voyage of the Beagle, vol. iii, p. 466.) In specimens which he dissected, he found the stomach loaded with minced sea-weed.

The teeth of the *iguanadon*, though resembling those of the iguana, do not present an exact magnified image of them, but differ in the greater relative thickness of the crown, its more complicated external surface, and, still more essentially, in a modification of the internal structure, by which the *iguanodon* equally deviates from every other known reptile.

As in the iguana, the base of the tooth is elongated, contracted, and subcylindrical; the crown expanded, and smoothly convex on the inner side. When first formed, it is acuminated, compressed, its sloping sines serrated, and its external surface traversed by a median longitudinal ridge, and coated by a layer of enamel, but, beyond this point, the description of the tooth of the iguanodon indicates characters peculiar to that genus. In most of the teeth that have hitherto been found, three longitudinal ridges (fig. 28) traverse the outer surface of the crown, one on each side of the median primitive ridge; these are separated from each other, and from the serrated margins of the crown, by four wide and smooth longitudinal grooves. The relative width of these grooves varies in different teeth; sometimes a fourth small longitudinal ridge is developed on the outer side of the crown. The marginal serrations, which at sight, appear to be simple notches, as in the iguana, present, under a low magnifying power, the form of transverse ridges, themselves notched, so as to resemble the mammilated margins of the unworn plates of the elephant's grinder: slight grooves lead from the interspaces of these notches upon the sides of the marginal ridges. These ridges or dentations, do not extend beyond the expanded part of the crown; the longitudinal ridges are continued further down, especially the median ones, which do not subside till the fang of the tooth begins to assume its subcylindrical form. The tooth at first increases both in breadth and thickness; it then diminishes in breadth, but its thickness goes on increasing; in the larger and fully formed teeth, the fang decreases in every diameter, and sometimes tapers almost to a

FIG. 28.

*Unworn tooth of Iguanodon.*

point. The smooth unbroken surface of such fangs indicates that they did not adhere to the inner side of the maxillæ, as in the iguana, but were placed in separate alveoli, as in the crocodile and megalosaur; such support would appear, indeed, to be indispensable to teeth so worn by mastication as those of the iguanodon. A fracture of this tooth shows that the pulp was not entirely solidified, but that its cavity had continued open at the thickest part of the tooth.

(To be Continued.)

## Figures and Tables

**Fig. 16. f1:**
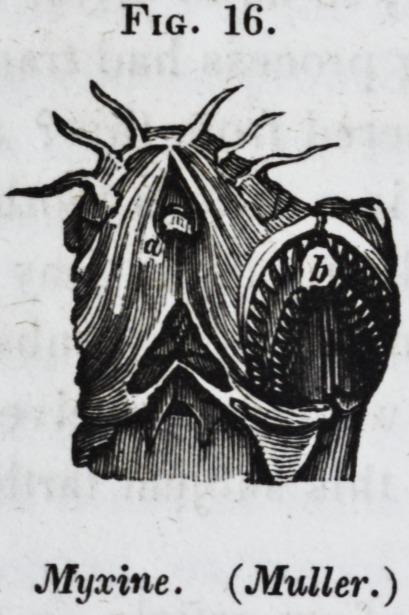


**Fig. 17. f2:**
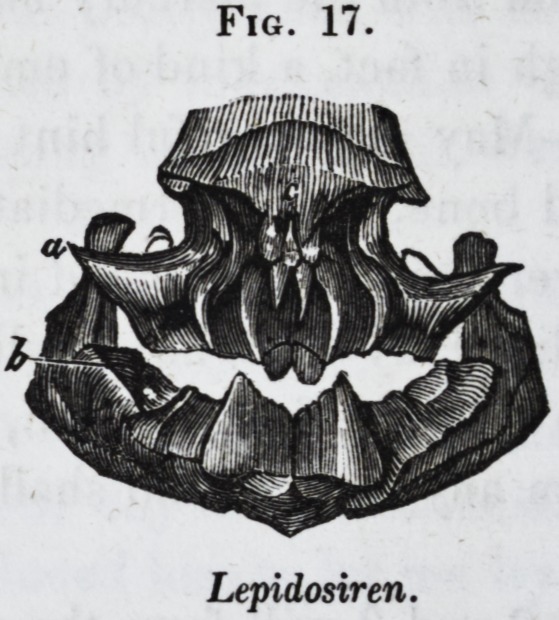


**Figure f3:**
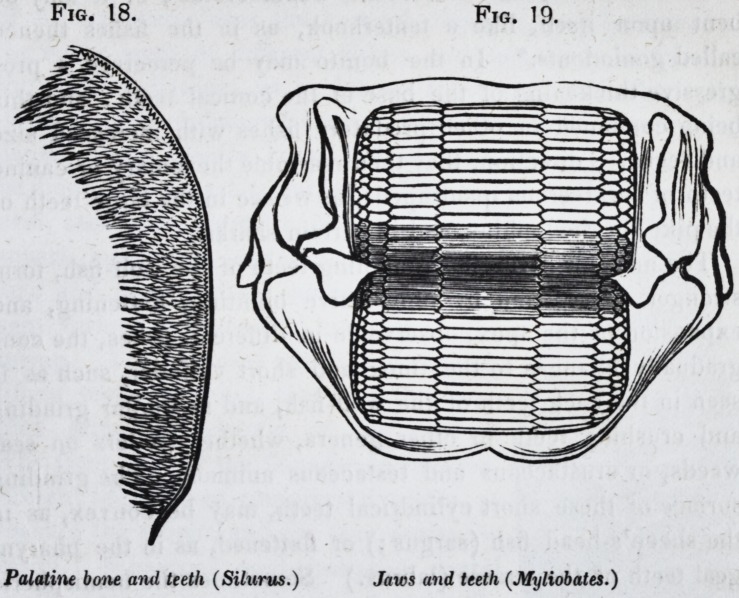


**Fig. 20. f4:**
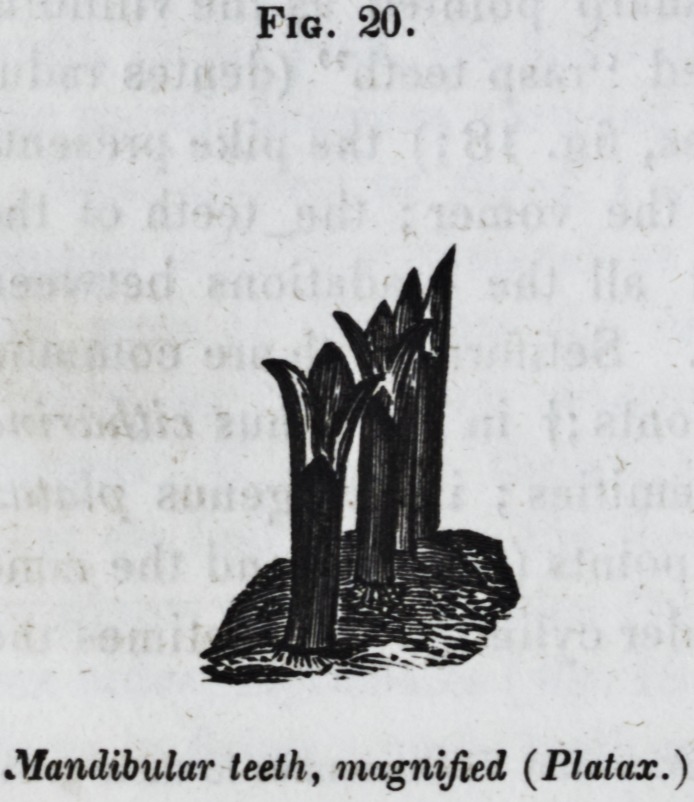


**Fig. 21. f5:**
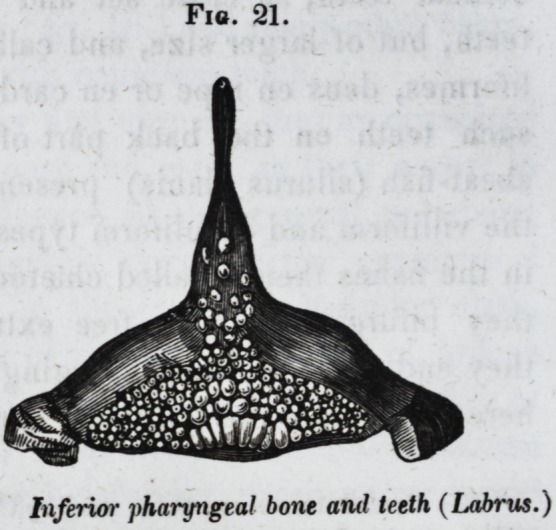


**Fig. 22. f6:**
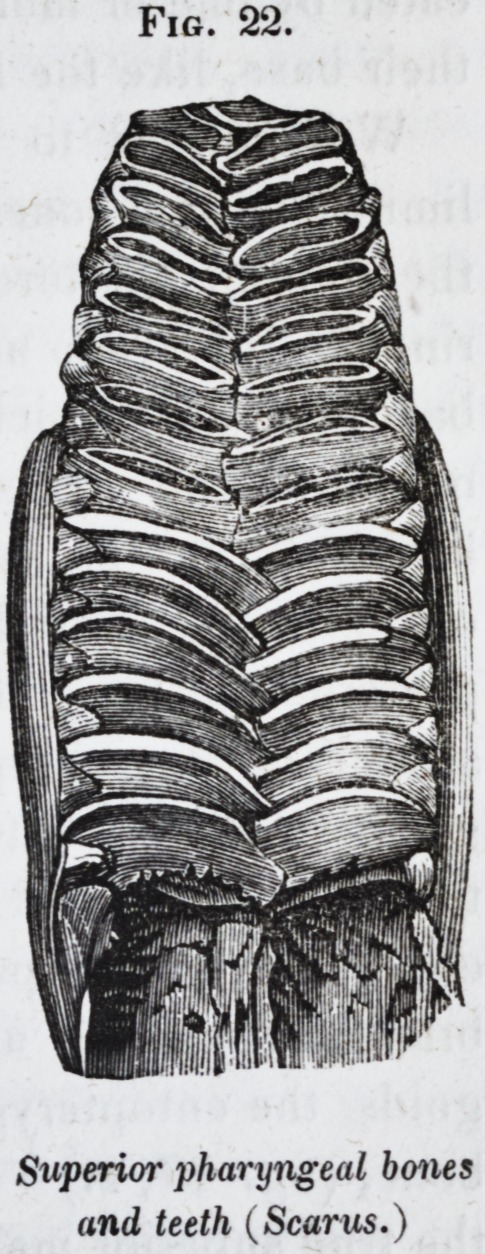


**Fig. 23. f7:**
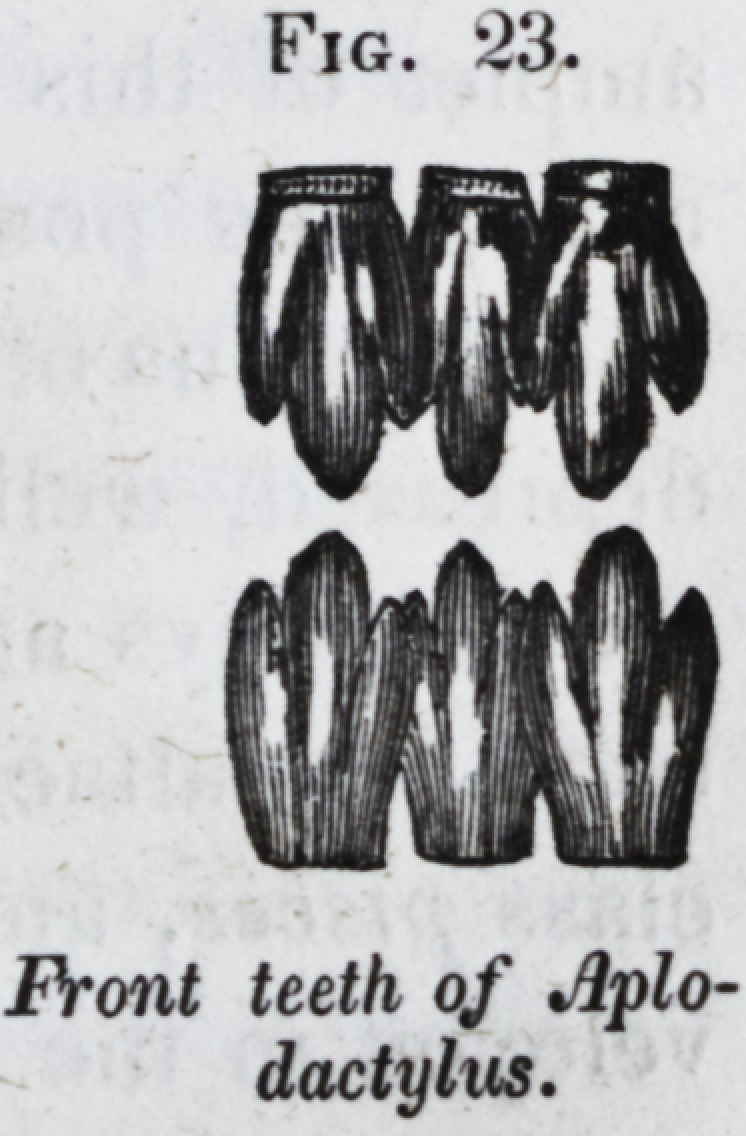


**Fig. 24. f8:**
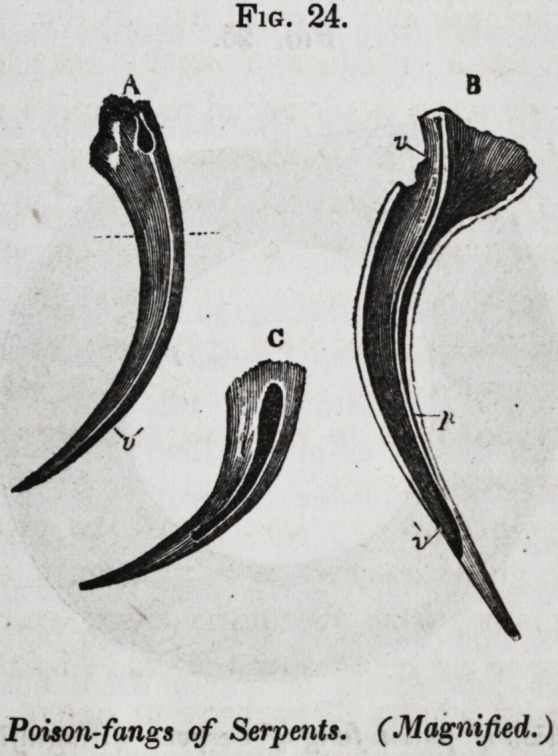


**Fig. 25. f9:**
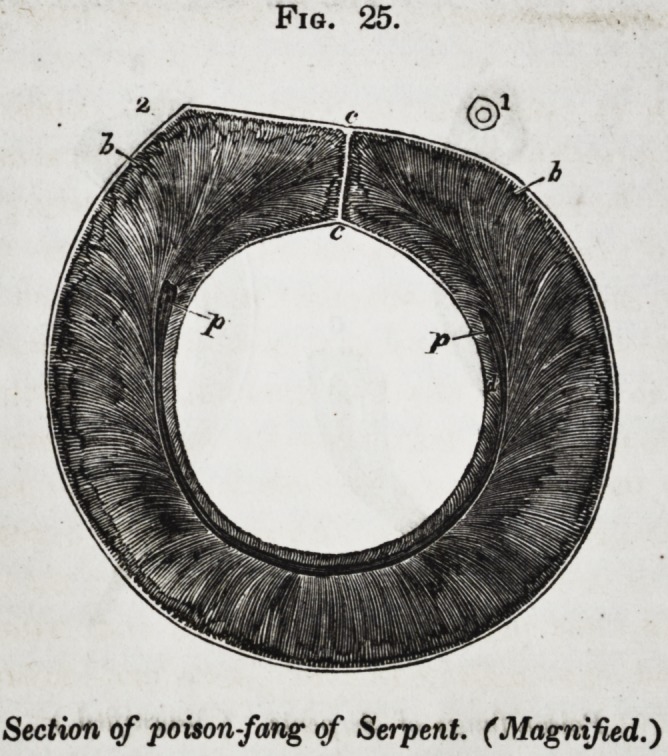


**Fig. 26. f10:**
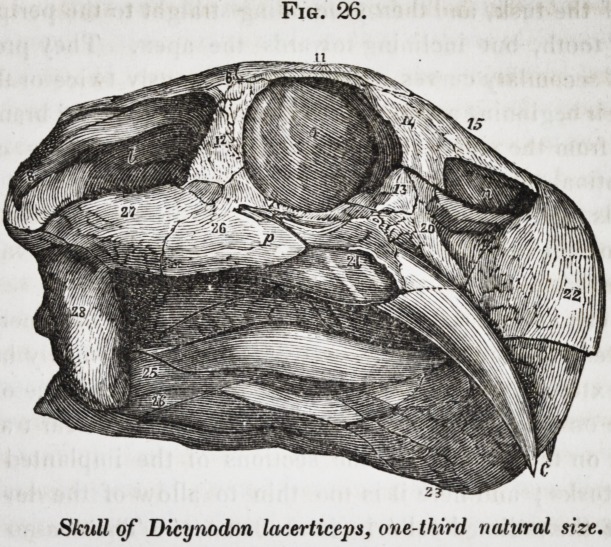


**Fig. 27. f11:**
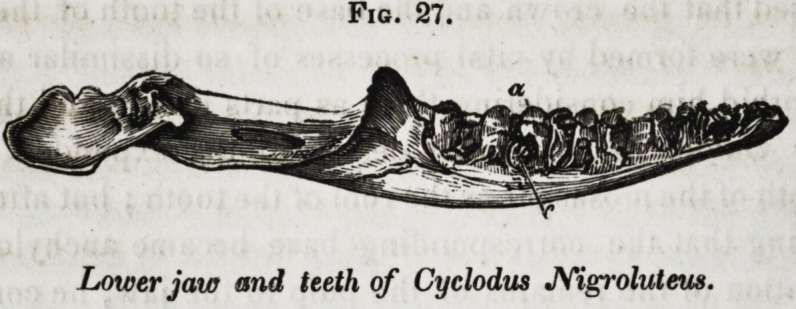


**Fig. 28. f12:**